# A Survey on the Feasibility of Sound Classification on Wireless Sensor Nodes

**DOI:** 10.3390/s150407462

**Published:** 2015-03-26

**Authors:** Etto L. Salomons, Paul J. M. Havinga

**Affiliations:** 1 Ambient Intelligence Group, Saxion University of Applied Science, P.O. Box 70000, 7500KB Enschede, The Netherlands; 2 Pervasive Systems Group, University of Twente, P.O. Box 217, 7500 AE Enschede, The Netherlands; E-Mail: p.j.m.havinga@utwente.nl

**Keywords:** wireless sensor networks, sound, context awareness

## Abstract

Wireless sensor networks are suitable to gain context awareness for indoor environments. As sound waves form a rich source of context information, equipping the nodes with microphones can be of great benefit. The algorithms to extract features from sound waves are often highly computationally intensive. This can be problematic as wireless nodes are usually restricted in resources. In order to be able to make a proper decision about which features to use, we survey how sound is used in the literature for global sound classification, age and gender classification, emotion recognition, person verification and identification and indoor and outdoor environmental sound classification. The results of the surveyed algorithms are compared with respect to accuracy and computational load. The accuracies are taken from the surveyed papers; the computational loads are determined by benchmarking the algorithms on an actual sensor node. We conclude that for indoor context awareness, the low-cost algorithms for feature extraction perform equally well as the more computationally-intensive variants. As the feature extraction still requires a large amount of processing time, we present four possible strategies to deal with this problem.

## 1. Introduction

Intelligent environments have been the subject of many experiments and research projects for the last few decades. We see projects in the context of healthcare and wellbeing that are often aimed at helping old and disabled people to remain independent in their own homes (e.g., Cook *et al.* [1], Kientz *et al.* [2]). Other projects aim to make the home environment more comfortable and to integrate entertainment appliances (e.g., Rashidi and Cook [3], Wu *et al.* [4]). A third category has its focus on improving productivity in office environments (e.g., Raskar *et al.* [5], Petzold *et al.* [6]). Finally, we see projects that deal with sustainability and energy usage reduction (e.g., Salomons *et al.* [7], Chassin *et al.* [8], Jahn *et al.* [9]). In this survey, we focus on intelligent home environments.

A requirement for all intelligent environments is the ability to perceive the conditions of the area in which the system is deployed and to do some form of reasoning and adaptation using the actuators that are present. A good method to gain awareness is by listening to sounds that are being produced by the environment. This has a number of advantages. First, sound sensors are non-obtrusive, although care has to be taken that the sound signals are not recorded, but are immediately processed instead. Second, sound waves are rich sources of information. Humans gain a large amount of context awareness through listening; they recognize voices or the specific gait of people they know, recognize the appliances that are being used through their specific sounds, determine the gender of people and the emotion of the verbal utterance and often recognize the environment by the sound of it (street, classroom, theater).

Context awareness that is based on sound is most effective when the sound is recorded at multiple locations in the area in which we are interested. This has a number of advantages. First, if we know the times of arrival of a sound event in three or more locations, it is possible to determine the location of the sound source. Second, a solution with multiple microphones allows filtering of unwanted background noise, such as television or radio sounds. A microphone that is installed close to these types of sources can determine whether or not the sound originates from this source and can supply sound parameters that can be used for compensation in the sound that is recorded in other locations. Third, if multiple devices individually process signals, they can switch roles when the need arises. For example, the node that is positioned close to a television in our previous example can be used as an extra input for localization when the television is switched off.

Ideally, we would like to have a high number of individual sensing and processing nodes. For newly built homes, a wiring plan for electricity and connectivity can be incorporated in the building plans. Existing homes do not have this possibility. To allow for a flexible solution that can be extended when the need arises without the need to install new infrastructures, the use of a wireless sensor network (WSN) is the logical choice. A WSN is easy to install or extend, and wireless nodes can easily be moved when the need arises.

A challenge when using WSNs is the limited processing power and working memory of the devices. If we want to use sound as the basis for context awareness for this type of device, we have to carefully select an appropriate algorithm for feature extraction of the signal and for training the system. Although there is ample research on context awareness using sound, this research in general has no limits with respect to the processing capabilities of the hardware. In order to make an appropriate choice for an algorithm, an overview of the strengths and weaknesses of the algorithms with respect to CPU load and the recognition rate is mandatory.

To meet this demand, we present a survey on the possibilities of using sound signals to gain context awareness on a WSN. Our approach is to look at this problem first from a high level point of view in order to gain insight into the possibilities and challenges when using this approach. This will help us to make the results applicable for a wider spectrum of devices and applications than would have been the case if we took a specific context as our starting point.

As sound processing on resource-limited devices is rarely the subject of research, we will first survey the use of sound for context awareness in general in Sections 2 and 3. Subsequently, we will focus on the applicability of our findings for wireless sensor nodes in Sections 4 and 5. Section 2 discusses the types of classifications that researchers are able to make using sound signals. Section 3 focuses on the methods of processing the sound signal or, to be more precise, on the feature extraction algorithms that are executed on sound signals in order to gain context awareness. In Section 4, we describe our efforts to harmonize the results of the literature in order to create a fair comparison of the performance of the features for the task of context awareness. Section 5 discusses the applicability of the feature extraction algorithms on wireless sensor nodes.

## 2. Categories of Sound Detection

There are several types of information that can be extracted from a sound signal. During our survey, we encountered seven categories: global sound classification, gender recognition, age classification, person recognition, emotion recognition, indoor environment sound source recognition and outdoor environment sound source recognition. These categories will be discussed in more detail in the following sections. There are other categories, but they are often too specialized to be of interest for our survey. An example of this is the classification of pig stress calls by Schön *et al.* [10]. Although Schöns results are interesting, there are no additional articles to be found on the same subject.

**Figure 1:**
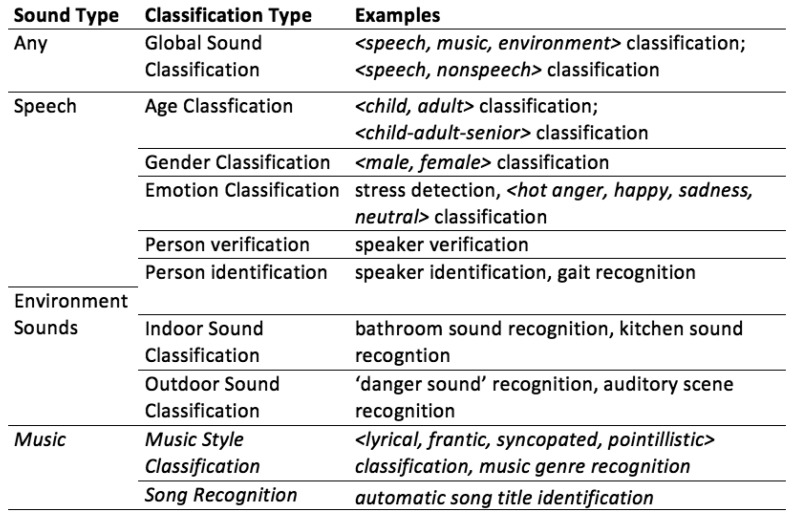
Classifications of sound events.

In Figure 1, we present an overview of the types of sound classifications that we see in literature. Most research projects are focused on recognition or classification of particular sound events; only a few projects deal with the challenge of automatically recognizing activities from sound signals, although this is, in many cases, the underlying reason for performing sound event recognition. Most closely linked to instantaneous activity recognition is the recognition of indoor sound events, as many indoor sounds can be linked to a single activity. We observe that, in general, the order and duration of the events are good indicators of the actual activities.

In this work, we will not look into the music subcategory in more detail. We are mainly interested in sounds that give information about the context. Although the music genre of background music can be an indicator of the environment, the number of cases in which this is actually useful is small.

### 2.1. Global Sound Classification

Global sound classification denotes the effort to classify the sound signal into a limited amount of classes that are dissimilar in nature. A typical example of the classes is <*music, speech, environmental sounds*>. Often, this type of classification serves as a preprocessing step for the actual application. For example, before speech recognition is performed, it is necessary to know whether speech is actually present in the sound signal.

As the sound signals of these classes are highly dissimilar in nature, the required amount of processing power for the classification can be kept relatively low (see Section 4.3).

### 2.2. Gender Classification and Age Classification

As is the case with global sound classification, gender classification and age classification are often used as a preprocessing step for another application. In particular, speaker identification and verification problems can benefit from the results of gender and age recognition. Li *et al.* [11] conclude that limiting the search space to speakers from the same gender considerably reduces the error rates for speaker verification and identification.

Age recognition is harder when the number of classes is high (see also Section 4.4). Although some research tries to classify up to seven classes, most publications limit themselves to two or three classes, typically <*child, adult*> and <*child, adult, senior*>, respectively.

In many cases, recognition of age and gender is performed in conjunction. Of these two, gender recognition achieves higher accuracies than age recognition. Chen *et al.* [12] note that first detecting gender is beneficial for experiments that detect age.

### 2.3. Person Recognition

The recognition of people using sound is being researched in many projects. Typically, the recognition is taking place using the voice signals of people. A notable exception is the research performed by Alpert and Allen [13], who perform recognition using the gait of people on stairs.

When writing about speaker recognition, different authors sometimes use the same word with different meanings. To clarify the discussion, Homayoon Beigi [14] discerns six types of speaker recognition:

- Speaker verification (authentication): The speaker has identified himself; the speech signal is used as a kind of password.
- Speaker identification: Identify who is talking.
- Speaker and event classification: Pooling similar audio signals into bins; an example is gender classification.
- Speaker segmentation: Retrieve the parts of a sound signal that belong to certain speaker; gives an answer to the question “who is talking when?”.
- Speaker detection: Detect one or more (specific) speakers in a stream of audio; this category encompasses segmentation, as well as identification/verification
- Speaker tracking: Track a specific speaker in an audio stream; other speakers are regarded as irrelevant.

In all cases, we aim to retrieve characteristics that are particular to an individual from a spoken utterance. In this survey, the category “person recognition” encompasses both speaker verification and speaker identification.

Another field where human speech is analyzed is speech recognition. Although at first sight, speech recognition and speaker recognition are dissimilar in nature, many authors note that the features that are helpful in speech recognition also give high success rates for speaker recognition (see Section 4.5).

### 2.4. Emotion Recognition

Besides the explicit message that is present in human speech, an import part of human speech is the implicit message that is conveyed. The implicit message, or the implicit part of the message, is usually characterized by the emotion of the utterance. The subject of automatic emotion recognition is thoroughly described in an article by Cowie *et al.* [15]. In their article, they mention the augmentation of human judgment (e.g., in lie detection), tutoring (knowing when the user becomes bored or irritated), alerting (a hospital patient in distress) and entertainment (toys that respond to their owner's mood).

### 2.5. Environmental Sound Recognition (Indoor)

The need for indoor sound event recognition often occurs in projects that are concerned with healthcare and ambient assisted living. The sounds that are produced in the various rooms of a house are good indicators of the activities that take place. A good example is given by Chen *et al.* [16], who perform recognition of bathroom sounds in order to detect the personal hygiene behavior of dementia patients. Another example is given by Stäger *et al.* [17], who perform quite well in recognizing typical kitchen sounds (average: 85% accuracy).

### 2.6. Environmental Sound Recognition (Outdoor)

There are two main applications for outdoor environmental sound recognition. One application is the detection of “danger” situations. Łopatka *et al.* [18] use the sound signal as reinforcement for a video surveillance detection systems. The other application is detection of the type of environment (e.g., <*train station, roadside, nature*>). Outdoor sounds convey much information about the location in which the sounds are recorded, as shown by Peltonen *et al.* [19].

## 3. Features for Sound Detection

Now that we have insight into the types of classifications that are performed on sound signals, we will look into the different ways of extracting information from the signals. There are many ways of extracting features from a sound wave. Some of them are computationally inexpensive, whereas others require high processing power. For certain categories, some features are better suited for recognition than others. Before showing an overview of the places where features are being used, we group the features into four categories, which will be elaborated in the remainder of this section: time domain features (Section 3.1), frequency domain features (Section 3.2), features inspired by voice production and perception (Section 3.3) and long-term features (Section 3.4). In Section 3.5, we discuss the complexity of these algorithms. The actual differences in calculation time during a benchmark test will be presented in Section 4.

More details about the calculation of the various features can be found in Appendix A.

### 3.1. Time Domain

Most feature extraction algorithms require a frequency analysis as the first step. There is however a small group of algorithms that use the signal in its raw form. These time domain features are often used when processing power is an issue. The preprocessing that needs to be done for this type of feature is less than when using frequency domain features. Applications that are deployed on wireless sensor nodes or on wearable devices often employ time domain features to gain knowledge about the environment. For these devices, battery-life is an important issue, so the algorithms that are being used must be computationally inexpensive.

Typical time-domain features include the following:

- The zero crossing rate (ZCR) is the rate of sign-changes along a signal or the number of times that the sound signal crosses the x-axis. This feature excels in separating voiced and unvoiced frames. Voiced frames, for example music waves, have high ZCR rates. Unvoiced frames, which often occur in environmental situations, show low zero crossing rates. The human voice contains both voiced and unvoiced parts.
- Short-time energy (STE) is a measure for the energy of the frames of a sound signal. This measure gives insight in the intensity and the variations in intensity of a sound signal. As an example, there are more silence frames in speech than in music.
- The sound amplitude (SA) provides information about the proximity or the loudness of a sound source.
- Peak detection (PD) is the detection of the points in time that a sound signal exceeds a certain threshold.

#### 3.1.1. Haar-Like Features

Haar-like filtering for sound signals is a technique introduced by Jun Nishimura and Tadahiro Kuroda [20]. The technique of Haar-like filtering is well established in the field of 2D face detection, where it is used to recognize particular parts of faces (Papageorgiou *et al.* [21]).

Nishimura and Kuroda have adapted this idea to apply for one-dimensional sound signals. A Haar-like filter ranges in length from two to 20 and consists of consecutive one's followed by a number of −1's, with possibly leading or trailing zeros or zeros between the series of one and −1. For each frame (length: 20–32 ms) of the sound signal, the products of the filters *h_m_* and consecutive parts of the signal *s* are added in order to obtain the filter value *x_m_* (see Equation (1)).

(1) $$x_{m}=\sum_{n=0}^{N}\left|\sum_{k=0}^{W_{\text{filter}}}h_{m}\left(k\right)s\left(n\cdot W_{Shift}+k\right)\right|$$

Nishimura and Kuroda note that for each filter *h_m_*, the value of *x_m_* is typically highest for signals that contain a large amount of a specific frequency. Some effort has to be made to find the appropriate filters for the type of sound to be recognized. It must be noted that Haar-like filtering, for 2D applications as well as 1D signals, must be regarded as a weak classifier. However, calculation of Haar-like features can be performed very efficiently.

### 3.2. Frequency Domain

The frequency domain offers a number of interesting features that can be used for sound analysis. In this section, we will briefly describe the simple features. ‘Simple’ in this case points to the fact that the values of the Fourier analysis of the sound signal are more or less used without post-processing. We discern the following features:

- Spectral centroid: The balancing point of the spectral power distribution; gives an indication of whether the signal contains more higher or lower frequencies.
- Bandwidth: The width of the range of the frequencies that the signal occupies; this is a measure for the flatness of the sound signal.
- Spectral roll-off: The frequency bin below which 93% of the distribution is concentrated; this is a measure of the skewness of the spectral distribution.
- Spectral flux: The average variation value of spectrum between the adjacent frames in a one-second window; this is typically highest for environmental sound, a bit lower for speech and even lower for music.
- Weighted phase deviation: Phase deviations of the frequency bins in the spectrum weighted by their magnitude; as an example, ambient sound and music will have a smaller phase deviation than voice.
- F0/Base frequency: A measure of the base formant of speech; this is often used in speaker recognition.
- Cochleogram/spectrogram: A graph that shows the presence of frequencies in the sound signal over time; this is often used in speech recognition.

### 3.3. Sound Production and Perception

Another method of extracting features from a sound signal is to look at the way sound is being produced by the human vocal system and how sounds are perceived by the auditory system. Using this inspiration, a number of methods of feature extraction have come up as valuable for speech recognition. Interestingly, these features are often suitable for speaker detection, as well. We consider two of the most popular types of features: linear predictive cepstral coefficients (LPCCs) and mel (melody) frequency cepstrum coefficients (MFCCs).

#### 3.3.1. Linear Predictive Cepstral Coefficients

**Figure 2:**
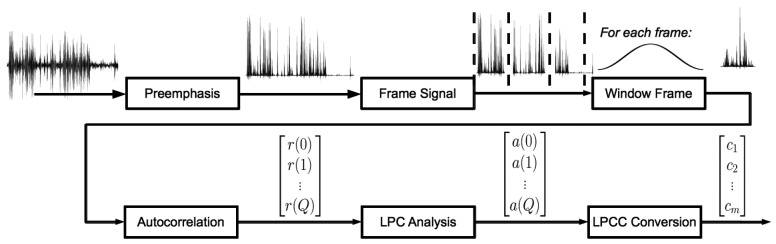
Linear predictive cepstral coefficient (LPCC) calculation.

Linear predictive analysis is inspired by the way that the human vocal system produces sounds. The method provides accurate estimates of speech parameters, such as pitch, formants and spectrum. LPC analysis was introduced in the late 1960s. The method analyzes the speech signal by estimating the formants of the sound wave through autocorrelation. Each sample of the original sound signal is expressed as a linear combination of the previous samples. Typically, the number of coefficients that is estimated ranges from 10 to 20. The coefficients are subsequently used to perform a cepstrum (inverse spectrum) analysis. The objective of this cepstral analysis is to isolate the contributions of the excitation source and the vocal tract system components. This process is graphically depicted in Figure 2. More details can be found in Appendix A.

#### 3.3.2. Mel Frequency Cepstrum Coefficients

**Figure 3:**
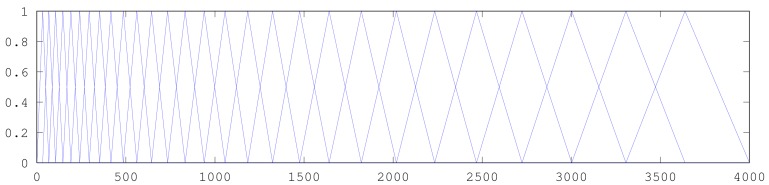
Mel (melody)-spaced filter bank.

**Figure 4:**
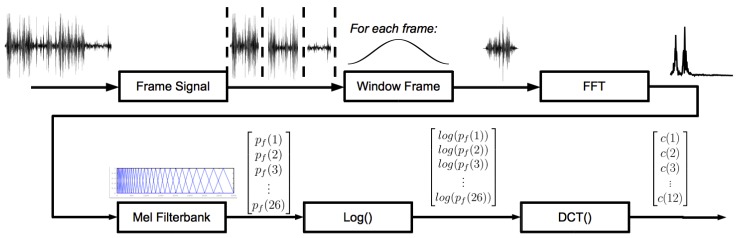
Mel frequency cepstrum coefficient (MFCC) calculation.

MFCCs are inspired by the human auditory system. Human perception of frequencies does not follow a linear scale. Variations in lower frequencies are perceived more accurately than variations in high frequencies. To take this into account, the subjective pitch is measured on the “mel scale”; see Figure 3. This scale is more or less linear for frequencies up to 1 kHz and logarithmic above this value. The coefficients are calculated by applying the mel scale to the results of the Fourier transform first. After this step, the discrete cosine transform is calculated. The amplitudes of the resulting spectrum constitute the MFCCs. Typically, the number of coefficients is 12. This process is graphically depicted in Figure 4. More details can be found in Appendix A.

### 3.4. Long-Term Features

The previous features all are calculated using small frames of the sound signal (20–40 ms). The last category of features are used to capture properties of the sound signal over a longer period of time. Two good examples of long-term features are jitter and shimmer. Jitter is defined as the variation in the base frequency of the sound signal; shimmer is the variation in amplitudes. These features are known to carry information regarding the age and gender of speakers.

### 3.5. Orders of Complexity

In order to gain a first notion of the algorithms' calculation cost, we consider their orders of complexity. See also Appendix A for more details about the calculation of the features.

- Time domain features (ZCR, STE, sound amplitude (SA), PD): *O*(*N*)
- Haar-like features: *O*(*N*); for each filter *h_m_*, the values are multiplied with the signal values *s*(*i*). If the number of filters is *f*, this results in *f* * *N* multiplications.
- Frequency domain features: Press et al. [22] argue that the FFT can be calculated in *O*(*Nlog*_2_*N*) complexity. All frequency domain functions require an additional *O*(*N*) step after calculation of the frequency spectrum, so they each have the same order of magnitude as the calculation of FFT.
- MFCC and LPCC both use the FFT as one of the main steps. Other steps that are performed in both algorithms have lower complexity than the calculation of the FFT. Therefore, the complexity of both algorithms can be expressed as *O*(*Nlog*_2_*N*).
- Jitter, shimmer: small additional calculations required after calculation of F0 and SA, respectively. Looks at consecutive frames; the order of this is *O*(*N*), so jitter and shimmer have orders equal to the FFT (*O*(*Nlog*_2_*N*)) and time domain (*O*(*N*)), respectively.

The orders of complexity give information about the asymptotic behavior of the algorithms. Constant factors are a negligible factor for higher numbers of data. We see proof for this in our benchmark tests (Section 4.1).

## 4. Performance of Features for Sound Detection

The previous sections give an overview of the types of classifications of sound signals and of the features that can be extracted from these signals. In this section, we connect these two categories. We will give insight into the achieved results of classification experiments that use the various types of feature extraction found in the literature. We will specifically look at the calculation costs and achieved accuracy of the various approaches.

For the recognition accuracy, we use the numbers that the authors provide in their articles. See Appendix B for a detailed description of the results found in the surveyed papers. The detailed description also provides insight into the training method that has been used in the respective papers. In this survey, our goal is to make a fair comparison of these papers. There is however a challenge as a result of the different ways that the results are being presented. Some authors measure recognition accuracy for their experiments, while others aim to find the equal error rate by tweaking the parameters of the learning algorithms. Some authors have detailed information about the classes that have been found, and others present their results in a more global way. Another major difference between articles is the number of examples that have been used for training and verification. As a result, some articles present more accurate results than others.

For the processing power metric, we calculate the relative execution time (RET), which we determine using a procedure that we describe in Section 4.1. For the experiments that combine features, we add the RETs of the separate features in order to estimate the combined calculation.

In Section 4.2, we briefly consider the memory footprint of the feature extraction algorithms that we use throughout this survey.

Sections 4.3 through 4.7 provide the results of our comparison. The graphs that we present use the two axes that we are interested in (RET *vs.* accuracy). The data points in the graphs show the features that have been used to achieve the presented accuracies and the authors and year of the concerning article.

### 4.1. Relative Execution Time

As each author uses their own implementation of the feature extraction algorithms, we wrote our own tests that compare the execution time necessary for extracting the different features. The times are compared with respect to the algorithm that takes the least time, hence relative execution time. In order to be able to compare the different features in a fair way, we implemented the test algorithms on an actual wireless sensor node: the Jennic JN5148 module. The microprocessor of this module consists of a 32-bit RISC CPU and has 128 kB available for program code and data. By choosing only one specific device to perform our benchmark test, we do not deliver extensive proof of the validity of the results for wireless sensor nodes in general. However, our choice for a representative hardware platform provides us with strong indications that these results are valid for a broad spectrum of WSNs. Devices that are equipped with hardware that is dedicated to a certain task of signal processing, such as hardware Fourier transforms, will have better performance on the benchmark tests that we performed.

The benchmark program is directly compiled for the Jennic JN5148 chipset. The nodes have no additional operating system. Besides the test software, no other processes are running. All calculations are performed using floating point numbers. All algorithms use the same sound fragments. These sound waves have a sampling frequency of 8 kHz, which is a frequency that is both feasible for the Jennic analog-digital converter and holds enough information of the recorded sound. For the calculation of the features, it is best to choose a frame length of 20–40 ms. If the signal is shorter, the number of samples for a spectral estimate will be too low; if the signal is longer, the signal changes too much throughout the frame, which causes the results of the feature extraction algorithms to become less meaningful. As the Fourier transform, which is used by the frequency domain features and MFCCs, requires a frame length equal to a power of two, we choose a frame length of 256 samples. This frame length corresponds to 32 ms, which fits in the desired interval of 20–40 ms. This frame length is used by all of our benchmark tests.

The RET is determined by measuring the time it takes for each feature extraction algorithm to finish. We noted that repeated executions of the benchmark tests resulted in exactly the same measurements, as did running the same test on two different nodes. The length of the sound wave that was processed had very little effect on the outcome of the measurements. The difference in processing time between a signal of one second and a signal of 32 ms was less than 0.1% for all algorithms.

**Figure 5:**
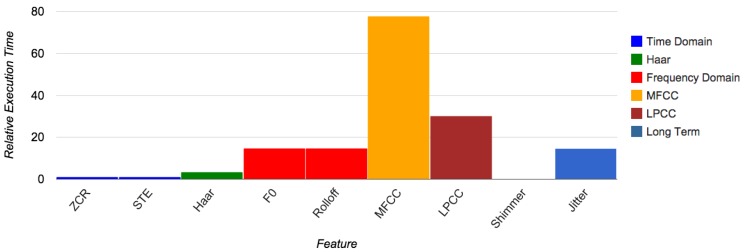
Relative execution time per feature. ZCR, zero crossing rate; STE, short-time energy; F0, base frequency.

Figure 5 shows the resulting RET for a subset of the different features that we tested. The features that are not present in this figure have computation times that are of the same magnitude as the other features of the same category. A notable difference exception to this rule are the long-term features (jitter and shimmer). Calculation of jitter is comparable to frequency domain features, whereas shimmers are calculated even faster than other time domain features. The reason for this is that the calculation of shimmers requires almost no multiplication operations.

Table 1 shows the RET for each group of features. We choose the value one for the least computationally-intensive feature category. As research that uses long-term features usually includes both jitters and shimmers, the value for this category is derived from the most computationally-intensive feature. The abbreviations in Table 1 will be used in the remainder of this article.

It might seem odd that the benchmark program of MFCCs results in a RET five-times the size of the corresponding value for frequency domain (FD) features, as both have the same order of complexity (see Section 3.5). This difference is caused by the higher number of steps that must be taken in order to calculate MFCCs (Section 3.3.2), including the calculation of 26 filter values and a discrete cosine transform. These steps have a constant length, independent of the frame size. In other benchmark tests, we saw that for longer frames (1 s and up), the RETs of the FD and MFCC algorithms are much closer. As we argued before, however, this is too long for meaningful feature extraction.

### 4.2. Memory Footprint

Besides RET, another metric that might be of interest is the memory footprint of the various feature extraction algorithms. In our implementations, the algorithms have between 10 and 200 lines of code. The memory demands are influenced most by the length of the frames of the sound signal that is being analyzed, as for some algorithms, it is necessary to allocate a buffer of the same size for intermediate results. These frames are short by design (see Section 4.1). In our benchmark programs, this resulted in a need for at most 1.6 kB of memory. Doubling the frame size (or the sampling rate) results in a demand of at most 2.6 kB. Given the fact that sensor nodes often have 100 kB or more of memory, these memory demands are not of great importance when considering which algorithms to use. Memory footprint is therefore not included as a criterion for the remainder of this survey.

### 4.3. Global Recognition of Sounds or the Classification of Sounds into Global Categories

**Figure 6:**
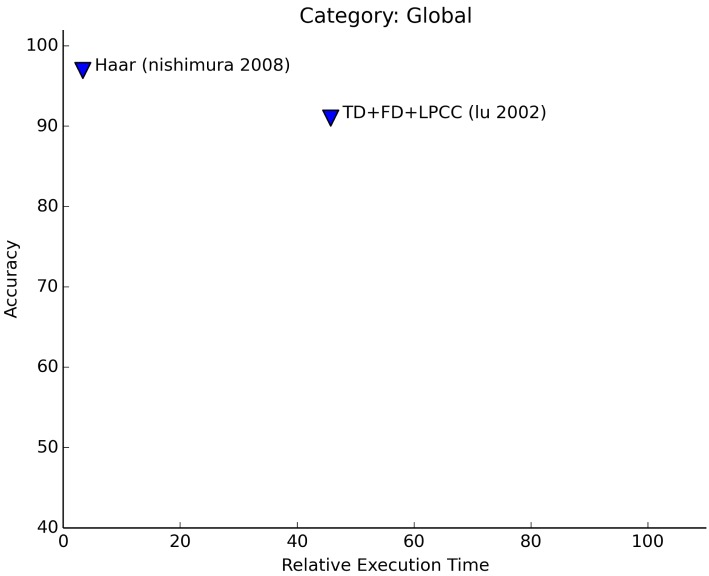
Relative execution time for global sound recognition.

The classification of sounds into global categories can be done with high accuracies (see Figure 6). Not many articles can be found that deal with this type of classification. The work that has been performed in this field shows that the classification into <*speech, non-speech*> classes can be performed with the highest accuracies using simple algorithms; speech consists of a narrower bandwidth of frequencies and has more pauses (silence) in the sound signal than non-speech signals.

As the signals are different in nature, the features that are chosen for the recognition task are not required to be highly discriminative for similar sounds. Instead, it is sufficient to use “lightweight” features, like time domain features and simple frequency domain features. The learning techniques that are used for this category have in common that they are aimed at finding similarities of individual samples (e.g., K-nearest neighbor, vector quantization).

In Figure 6, we see that the required processing power for the algorithms of both articles is quite low. Nishimura and Kuroda [20] only use the computationally-efficient Haar-like features to distinguish speech from non-speech sounds. Lu *et al.* [23] also take into account the music category. Using only simple time and frequency features, they manage to achieve a high separation between speech and non-speech sounds (approximately 95% accurate). The other categories are harder to distinguish using only these types of features. Using linear spectral pairs for the experiments improves the results of the recognition of music (93% accuracy) and other environment sounds (84% accurate).

### 4.4. Gender and Age

**Figure 7:**
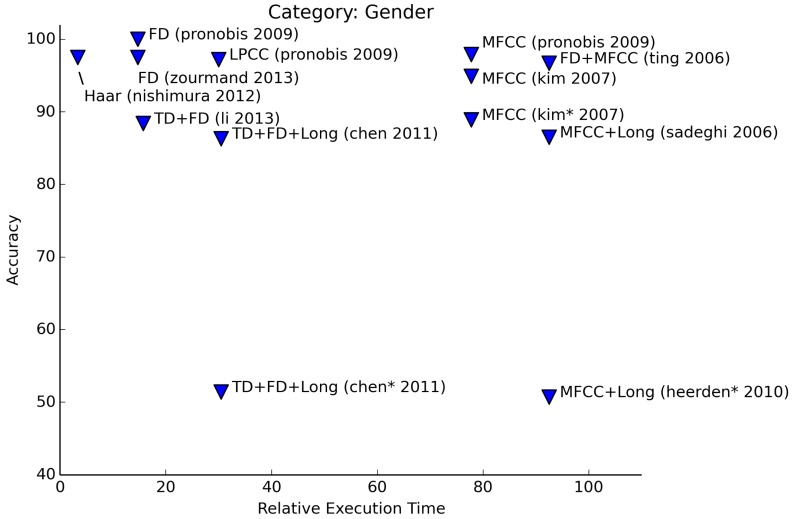
Relative execution time for gender recognition.

In Figure 7, we see that the detection of the gender of a speaker can be done with high accuracy. Apart from time domain features and long-term features, most features are suitable for gender recognition. For most authors, there is even no need to combine features in order to increase accuracy. Pronobis and Magimai-Doss [24] show that the F0 feature and higher order features (MFCC, LPCC) perform comparably well. F0 is a value that denotes the base frequency of the sound signal. This corresponds with the way people distinguish male voices from female voices: men have lower voices than women. Haar-like features can be selected to be responsive to certain frequencies, so basically, this leads back to the same principle. Pronobis and Magimai-Doss have a spectacular 100% accuracy for gender classification using only F0. This perfect score is only achieved when classification is performed on the voiced speech parts of a clean speech signal. Experiments that use all speech frames or degraded sets of speech signals perform slightly worse, although the accuracy remains higher than 93%.

**Figure 8:**
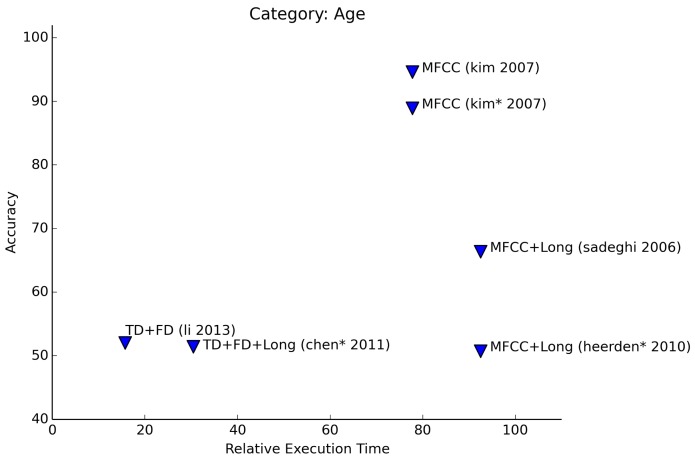
Relative execution time for age recognition.

Estimation of age results in lower accuracies than recognition of gender (see Figure 8). Experiments that are limited to the distinction between adults and children achieve the best results. Finer-grained distinctions lead to estimates that are sometimes only a little better than random guessing. A good example of the accuracy getting lower is given by Sadeghi and Homayounpour [25]. For two age classes, the accuracy is 72%, and one more added class causes the accuracy to drop to 61%.

In three articles, the authors describe experiments to determine age and gender simultaneously. In Figures 7 and 8, these results are marked with an asterisk (*). Of these results,Kim *et al.* [26] manage to get the highest accuracy. In their paper, they describe experiments to determine both the age and gender of a person separately, as well as simultaneously. Age is restricted to the classes <*adult, child*>. The age + gender experiments involve the classes <*male, female, child*>. Chen et al. [12] perform recognition of age and gender in two stages: first, gender is determined with a good accuracy (91% male, 81% female). The second stage consists of the determination of the age group (<*child, adult, elder*>). Performance drops significantly in this case (54% accuracy), although age determination without the previous step of gender recognition delivers even lower accuracies. Van Heerden et al. [27] even include a fourth age group (<*child, young, adult, senior*>). The performance of their experiments are comparable to Chen et al.

### 4.5. Identification of People

One of the most frequent uses of sound classification in the literature besides the recognition of speech is the identification of people. Two types of features are dominantly used for this purpose. Nowadays, many projects use MFCCs, having more or less replaced the usage of derivatives of linear predictive coding. Although the inspiration for both types of features is different, the results are more or less comparable. In some cases, LPCCs outperform MFCCs; in other cases, MFCCs are better.

The results in the papers we considered are often hard to compare, as the number of speakers that are to be identified or verified varies highly. Nevertheless, it can be interesting to look at the differences. We see, for example, that the results for identification attempts are usually more accurate than the results for the verification attempts. This can be counter-intuitive at first sight. From a human perspective, it seems to be harder to determine the speaker's identity than to confirm or refute the identity. However, given a limited amount of possible speakers, it is actually easier to look for the person whose voice is most similar to a given utterance. For verification, the system has to be almost certain that the speaker is indeed the right one. This test set should therefore be more varied than the test set for identification.

Although MFCCs and LPCCs are used most often, the papers of Alpert and Allen [13] and Kinnunen *et al.* [28] are worth mentioning. Alpert and Allen only use the intensity of the sound signal for gait recognition on staircases. In the sound signals, the peaks are determined. Using these peaks, the typical up- and down-stairs walking patterns of inhabitants of a home are used for training a neural network. In their experiments, they achieve up to 90% accuracy over a set of four actual household inhabitants. Interestingly, there are differences in recognition rates between inhabitants going up the stairs and inhabitants going down. The down-going gait appears to be more consistent than the up-going gait. Kinnunen *et al.* compare the usage of frequency spectrograms with MFCCs. For the frequency approach, they developed a dimension reduction technique that resulted in a verification success rate of 83%. The same task, when performed using MFCCs, resulted in an accuracy of 93%.

Four papers focus on identification of persons: Nishimura [29], Alpert and Allen [13], Hasan *et al.* [30] and Kim *et al.* [31]. We see, that the accuracy of these experiments is a bit higher on average than the other experiments that focus on speaker verification. The work of Kim *et al.* uses a high number of speakers for their experiments (195); the others have a more limited amount of people to identify (up to 24).

The other authors focus on speaker verification. The number of speakers in these experiments is rather high for most papers (over 100), with the exception of Tiwari *et al.* [32] and Reynolds *et al.* [33], who only have a small dataset of persons to be verified.

**Figure 9:**
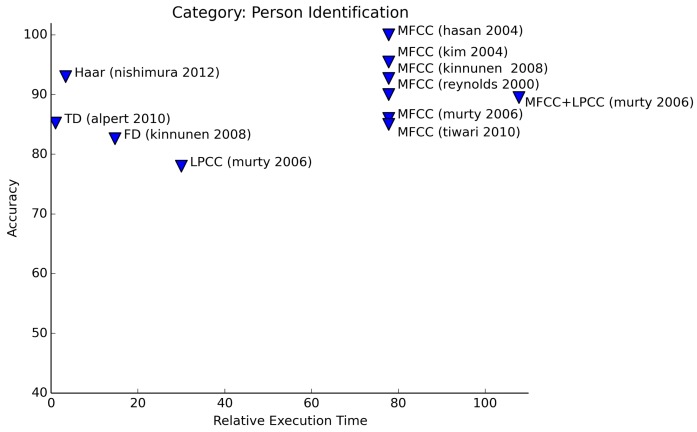
Relative execution time for person recognition.

From the results of Figure 9, we may conclude that the safest option for person recognition (whether identification or verification) is to use MFCC features. Another good choice, or even better if only looking at the necessary processing power, is to use Haar-like features. As we already saw in Section 3.1.1, Haar-like features are in fact representatives of certain frequency components.

### 4.6. Emotion

**Figure 10:**
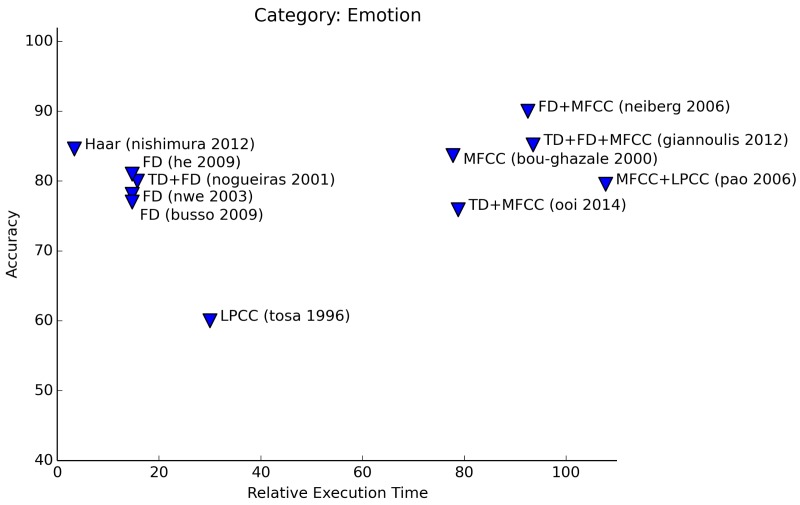
Relative execution time for emotion recognition.

Emotion recognition is another widely surveyed subject (see Figure 10). The number of emotions that authors are attempting to identify varies highly. The table data show that it is hard to identify an emotion if the amount of emotions is higher than three. Projects that attempt to classify three emotions (e.g., <*happy, sad, neutral*>, Nishimura [29], Neiberg *et al.* [34]) or that perform stress detection (He *et al.* [35]) are usually able to do so with high accuracy. Attempts to classify more than three emotions see the accuracy rate drop. Higher-order algorithms for the extraction of features do not raise the performance of recognition significantly. As an example, both Nwe *et al.* [36] and Pao *et al.* [37] aim to classify emotions in one of six classes and have results that are, on average, equally good. The latter extracts MFCC features from the sound signal; Nwe *et al.* use the log frequency power coefficients that are derived from the Fourier analysis. Other authors successfully use simple frequency features, as well. Busso *et al.* [38] and Nogueiras *et al.* [39] rely on pitch aspects of the sound signal.

One type of learning technique for emotion recognition is similarity-based (Gaussian mixture model, artificial neural network). Another technique that is often used is the hidden Markov model (HMM). The latter can be a logical choice, as the HMM takes into account the history and order of a sound signal. Many emotions can be characterized by order. As an example, both crying and laughing consist of a number of short bursts of sound, often followed by a brief pause.

### 4.7. Environment

**Figure 11:**
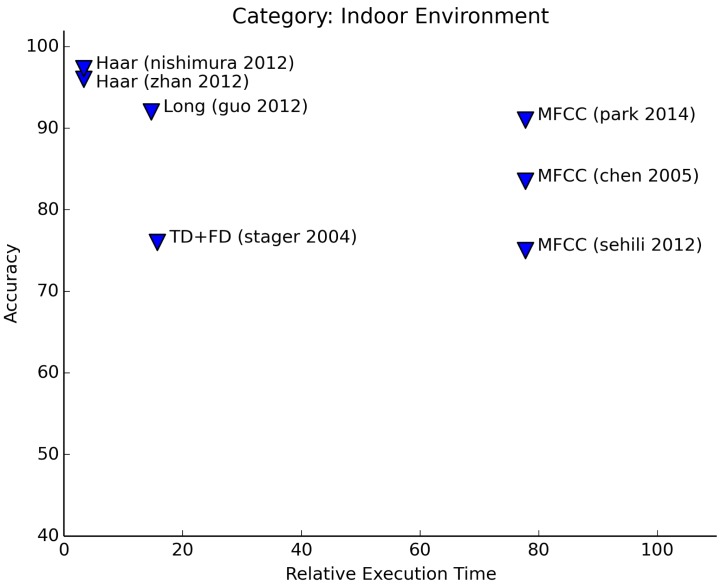
Relative execution time for indoor environment recognition.

**Figure 12:**
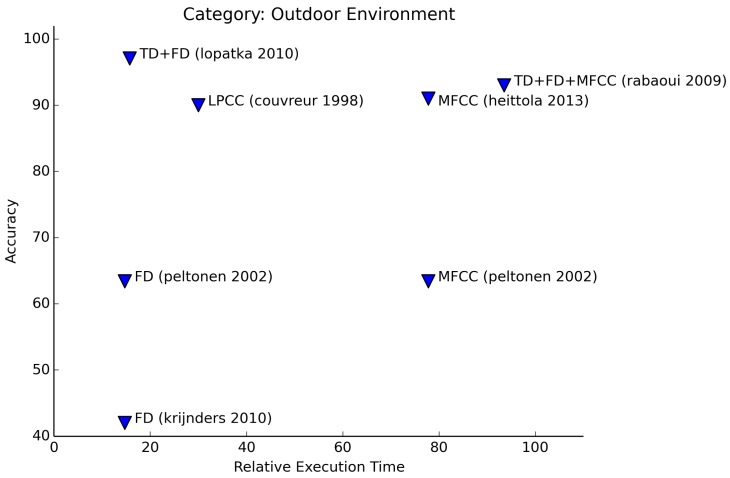
Relative execution time for outdoor environment recognition.

Although the application of indoor and outdoor environment recognition is quite different, the features that are being used for both and the applied learning techniques are often similar (see Figures 11 and 12). This could be expected, as both indoor and outdoor environment sounds are noisy in nature. For this category, the amount of sounds to be recognized varies highly between authors. Both authors that use Haar-like features (Nishimura [29] and Zhan [40]) manage to achieve high accuracies (95%+) for recognition of 20+ office sounds. This is better by far than other experiments. Łopatka et al. [18] achieve a similar accuracy on the recognition of only five sounds. These sounds are all from the domain of danger sounds.

Just as with emotion recognition, we see similar results for using frequency features and MFCCs.

Although for some sounds, it seems enough to recognize the particular sound features, for some events, performance is higher when applying an HMM for learning. Apparently, the order of the sound events carries some essential information.

## 5. Sound Features for Wireless Sensor Nodes

Now that we have gained insight into the methods of context inference using sound, we will assess these methods with respect to the feasibility of their application on a resource-constrained platform, such as a wireless sensor node. In particular, we aim for algorithms that have low CPU time and high accuracy for this particular task.

**Figure 13:**
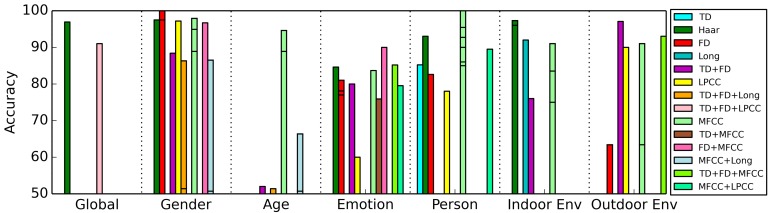
Overview of feature comparisons, grouped by category.

An overview of the cost and effectiveness of the different types of feature extraction algorithms for the task of context recognition is presented in Figure 13. The features are grouped by category and are ordered from low to high RET within each group. Multiple horizontal lines in a bar indicate that different authors have found different accuracies using this feature. As an example, we found three authors that use MFCCs for gender recognition. We see that gender recognition, emotion recognition and person recognition are popular subjects of research. Gender recognition and person recognition especially yield high accuracies, although for each category, accuracies higher than 90% are achieved.

**Figure 14:**
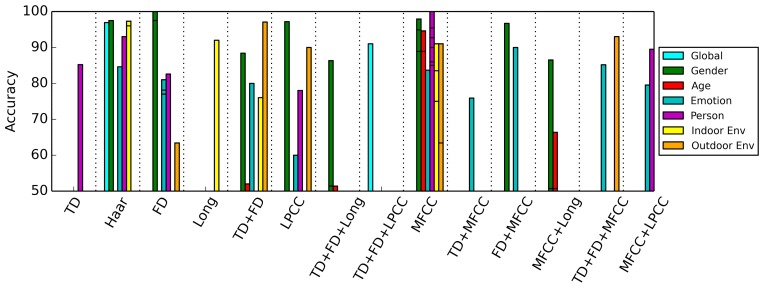
Overview of feature comparisons, grouped by feature.

The results can also be grouped by feature. Figure 14 shows the results of this effort. In this figure, we see for which applications a certain combination of features is appropriate. What draws attention is that Haar-like features and MFCCs are widely applied and yield high accuracies for a number of applications.

One conclusion that can be drawn is that there is no silver bullet for this task. The results in Section 4 show that many types of context can be equally well identified with different sets of features. That is of great interest if we want to apply these features on wireless sensor nodes. For this particular platform, we want to use algorithms that have a low calculation cost in order to accommodate the restricted resources available and the limited battery-life of these devices.

Although the experiments that are described in the papers that we reviewed are generally performed on hardware platforms that have ample resources, we learned through our benchmark tests that these results can be replicated on resource-constricted hardware, such as wireless sensor nodes; that is, taking into consideration that the feature-extraction algorithms and learning algorithms can be executed within acceptable time. A good example of a resource-friendly training method is given by Marin-Perianu *et al.* [41]. The work of Nishimura [29] and Zhan [40], who employ Haar-like feature extraction, is promising in particular for feature extraction, as they execute their algorithms on wearable devices. The achieved high accuracies and low calculation costs make this a good candidate for this type of application. Surprisingly, it is not widely in use yet. This may be due to the fact that many research projects do not focus on wireless sensor nodes and, therefore, do not have this limitation in processing power.

In Section 2, we considered a number of categories for gaining context awareness. When we restrict ourselves to an indoor setting based on wireless sensor nodes, not all of these categories are equally feasible. Person identification or recognition has constraints that reach beyond what can be done on resource-constrained nodes, not only because of the cost of feature extraction, but also because of the large amount of data that has to be used for training and deployment. Given the low-cost nature of the sensors that we want to deploy, it is not possible to achieve a high accuracy of person recognition. For the same reasons, emotion recognition falls out of scope.

What remains of interest are global recognition, gender and age recognition and indoor environment recognition. As the calculation of the MFCC features takes too much processing power in comparison to the other features, we will consider alternative approaches as outlined below.

### 5.1. Global Sound Classification

Global sound classification can be applied to our setting as the first step to gain context awareness. Knowing that someone is speaking can trigger a module that is specifically aimed at age and gender recognition. Detection of music gives information about the activities that the people that are present are engaged in, and detection of environmental sounds can trigger the module that is developed for that part.

We see that the use of Haar-like features can be suitable for this situation. One problem with Haar-like features is the definition of the correct filters. More research is necessary in order to make a statement about the generality of this approach. We do see that the use of time domain features, frequency domain features and linear predictive coding leads to high accuracies. Therefore, the task of global sound classification can be very well employed on wireless sensor nodes.

### 5.2. Age and Gender Recognition

Gender recognition can be achieved with high accuracies using minimal CPU power. Simple frequency domain features are suitable, as well as employing Haar-like features. Age recognition is less accurate using low-cost features. This may be partly due to the number of classes that the various researchers are trying to recognize. The recognition of the <*male, female, child*> classes can be done with low RET and acceptable recognition rates. The male speaking voice typically has a fundamental frequency between 85 Hz and 155 Hz. The typical range for female speakers is between 165 Hz and 255 Hz, and a child's voice ranges from 250 Hz–600 Hz, or even higher for very young children. Because these frequency ranges are nearly mutually exclusive, fundamental frequency is a good indicator for these classes. This is not 100% accurate, as there are men with high voices and women with low voices, often due to a smoking habit.

### 5.3. Indoor Sound Recognition

The results of indoor sound classification indicate that these features are also highly dependent on frequency-like features. The addition of time-domain features enhances these results. Again, the Haar-like features achieve high results. A combination with higher-order features, such as LPCCs, might be something to take into consideration for this type of recognition.

## 6. Conclusions

Using sound for the purpose of gaining context awareness is the subject of many research projects. Even though the use of wireless sensor nodes seems logical for sound collection and processing, this area is not widely studied. This is partly due to the currently popular algorithms for feature extraction that are computationally intensive.

If we consider an indoor environment, we see that the classification of sounds into global categories can be performed with very low calculation effort (see Section 4.3). For gender recognition (Section 4.4), algorithms that use the low cost Haar-like features or frequency domain features achieve results that are comparable or better than algorithms that uses the heavy-weight MFCCs for classification. For specific indoor sounds (Section 4.7), the use of Haar-like features or long-term features achieves similar results compared to the use of MFCCs. We see that for these three categories (global, gender and indoor sound classification), the use of low-cost algorithms can be equally effective for deployment indoors as the use of high-cost algorithms.

One factor that we did not yet take into account is the challenges of training the learning algorithms for the recognition tasks. As we mentioned before, there are papers dedicated to these tasks. Future work will look into making these learning algorithms applicable for the task of context recognition based on sound.

There are still some challenges to solve if we want to apply our knowledge on feature extraction. A major challenge is the time it takes to extract the features from a sound signal. As an example, we consider using the Jennic JN5148 platform that we mentioned before and use it to capture sound at 8 kHz. For this setup, the time to calculate even the least computationally-intensive features approaches the sampling time of the signal itself. If we want to use frequency features, the processing time will be approximately 15-times as long as the sampling time.

We see a number of possible ways to deal with this challenge:

- Accept the long processing time: In many cases, there is no need to record sound continuously. For many events, the characteristics remain constant for a certain period of time. In these cases, it is not problematic if not all sound is recorded. If the nodes are able to capture the ‘interesting’ frames based on some heuristic (see, for example, Le *et al.* [42]), the remaining time can be spent on processing the sound waves.
- Lower the sampling rate: In the literature, a sampling rate of 8 kHz or higher is common practice, which allows one to effectively detect frequency formants of up to 4 kHz. For some applications, the frequency range that we are interested in is much lower than this. As an example, for gender recognition based on the fundamental frequency, we only need frequencies up to 600 Hz.
- Delegate tasks to dedicated nodes: One strategy is to have one node record sounds and transmit these sounds to several other nodes that are responsible for a particular part of the feature extraction and training. Sensor fusion techniques, such as proposed by Bahrepour *et al.* [43], can be useful for this approach. Another possibility is to synchronize a group of nodes. One node records sound for a limited time. After this period, another node starts recording while the first node is processing the sound. In this way, the lag between consecutive frames that can be processed is lowered proportional to the number of nodes for this task.
- Switch to more powerful hardware: If none of the above solutions is satisfactory, this might be the only option. There are a number of small and powerful devices commercially available currently, such as the Raspberry Pi, the BeagleBone Black and the Intel Edison. The downside of using this type of device is that they are less energy efficient. If we want to deploy these devices in a home, they are best deployed near electrical outlets.

In this article, we have not considered the payload of learning algorithms that use the features to infer context knowledge. It is not required that these algorithms have low payload during the training phase, as long as they are efficient during the production phase. These aspects will have to be carefully considered in future work.

## Figures and Tables

**Table 1:** Relative execution time (RET).

**Feature**	**Abbreviation**	**RET**
Time domain features	TD	1
Haar-like features	Haar	3.5
Frequency domain features	FD	15
Long-term features	long	15
LPCC features	LPCC	30
MFCC features	MFCC	78

## Appendix

### A. Feature Algorithm Details

Here, we present in more detail how the different features that have been discussed in Section 3 are calculated. In our formulas, we use *s*(*n*) to indicate the values of the original sound signal, *N* for for the number of sound samples, *p*(*f*) to indicate the power spectrum value for a certain frequency and *F* for the number of Fourier values.

#### A.1. Time Domain

- ZCR:
  (A1) $$\text{ZCR}=\frac{1}{2\left(N-1\right)}\sum_{n-1}^{N-1}\left|\mathrm{sgn}\left(s\left[n\right]\right)-\mathrm{sgn}\left(s\left[n-1\right]\right)\right|$$
  *sgn* is the signum function.
- STE:
  (A2) $$STE=\sum_{n=0}^{N-1}s{\left(n\right)}^{2}$$
- Sound amplitude:
  (A3) SA=max|s(n)|
- Peak detection/peak location:
  (A4) $$PL=\underset{n}{\text{argmax}}|s\left(n\right)|$$

#### A.2. Frequency Domain

Frequency analysis depends on the Fourier transform, which is calculated using the discrete Fourier transform:

(A5) $$H_{f}=\sum_{k=0}^{N-1}s\left(k\right)e^{-i2\pi f/N}$$

The calculation of the discrete cosine transform (DCT) is done using a fast Fourier transform algorithm. The power spectrum *p*(*f*) is calculated as follows:

(A6) p(f)=Re(Hf)2+Im(Hf)2

- F0:
  (A7) $$F0=\underset{f}{\min}\left\{f|p\left(f\right)>\theta \wedge p\left(f-1\right)<p\left(f\right)<p\left(f+1\right)\right\},$$
  where *θ* is a threshold value. Typically, *θ* = 0.1 Σ*_f_ p*(*f*).
- The spectral centroid for a frame can be defined as:
  (A8) $$SC=\frac{\sum_{f}p\left(f\right)*f}{\sum_{f}p\left(f\right)}$$
- The spectral roll-off for a frame can be defined as:
  (A9) $$RO=\underset{n}{\text{argmax}}\sum_{f=1}^{n}p\left(f\right)\leq \alpha \cdot \sum_{f=1}^{F}p\left(f\right),$$
  where *α* is a constant with a typical value of 0.97.
- The bandwidth is defined as:
  (A10) $$BW=\sqrt{\frac{\sum_{f}{\left(f-SC\right)}^{2}*p{\left(f\right)}^{2}}{\sum_{f}p{\left(f\right)}^{2}}}$$
- The weighted phase deviation is defined as:
  (A11) $$\text{WPD}=\underset{f}{\text{∑}}p\left(f\right)*\phi^{\prime \prime }\left(f\right)$$
  where *ϕ*(*f*) is the phase of the Fourier value for frequency *f*

#### A.3. MFCC

The MFCCs are calculated using the following steps:

1. Framing the signal: the signal is segmented into overlapping frames. The width of the frame is generally about 30 ms with an overlap of about 20 ms.
2. Windowing: A window function is used to smooth the signal for the computation of the DFT so as to minimize the signal discontinuities at the beginning and end of each frame. A typical window is the Hamming window, which has the form:
  (A12) $$w\left(n\right)=0.54-0.46\cos\;\left(\frac{2\pi n}{N-1}\right)$$
  The windowing is calculated using:
  (A13) $$\tilde{s}\left(n\right)=s\left(n\right)w\left(n\right)$$
3. FFT: The power spectrum *p*(*f*) of the windowed function *s̃*(*n*) is calculated as described in Equations (A5) and (A6).
4. The mel filter banks (see Figure 3) are applied to the power spectrum. The filter banks are evenly distributed over the frequencies with respect to the Mel scale, which is defined as:
  (A14) $$\text{Mel}\left(f\right)=2,595\log_{10}\;\left(1+\frac{f}{700}\right)$$
  The number of filters is 20–40; 26 is standard. This leaves us with 26 numbers that indicate the energy in each filter bank.
5. Take the log of the energies from the previous step.
6. Take the DCT of the 26 log filter bank energies. This is calculated by the following,
  (A15) $$c_{d}=\frac{1}{M}\sum_{m=0}^{M-1}C_{m}\cos\;\left(\frac{\pi \left(2d+1\right)m}{2M}\right)\;,$$
  where *c_d_* is the *d*-th cepstral coefficient, *M* is the total number of filter banks and *C_m_* denotes the log energy for filter bank *m*. Typically, *c*_1_ − *c*_12_ constitute the MFCCs.

#### A.4. LPCC

The LPCC are calculated using the following steps:

1. Pre-emphasis: The speech signal is spectrally flattened to make it less susceptible to finite precision effects:
  (A16) $$\tilde{s}\left(n\right)=s\left(n\right)-\tilde{a}s\left(n-1\right),$$
  where the filter coefficient *ã* is a constant with a typical value of 0.97.
2. Framing and windowing the signal: this is analogous to Steps 1 and 2 of MFCC calculation.
3. Autocorrelation analysis: this can be performed efficiently by calculating the inverse Fourier transform of the signal's power spectrum.
4. LPC analysis: Each frame *r*(*q*) of *Q* + 1 autocorrelations (with *Q* the order of the LPC analysis) is converted into an LPC parameter set using the Levinson−Durbin method:
  (A17) $$E^{\left(0\right)}=r\left(0\right),\alpha^{\left(0\right)}=1$$
  for (q = 1 to Q),
  (a) (A18) $$k_{q}=\frac{r\left(q\right)-\sum_{j=1}^{q-1}\alpha_{j}^{\left(q-1\right)}r\left(q-j\right)}{E^{\left(q-1\right)}}$$
  (b) (A19) $$\alpha_{q}^{\left(q\right)}=k_{q}$$
  (c) for (j = 1 to Q):
    (A20) $$\alpha_{j}^{\left(q\right)}=\alpha_{j}^{\left(q-1\right)}-k_{q}\alpha_{q-j}^{\left(q-1\right)}$$
    endfor:
    (A21) $$E^{\left(q\right)}=\left(1-k_{q}^{2}\right)E^{\left(q-1\right)}$$
  endfor.
  Once the above recursion is completed, the LPC parameters are extracted as follows,
  (A22) $$a_{q}=\alpha_{q}^{\left(Q\right)}$$
5. LPC parameter conversion to cepstral coefficients: The cepstral coefficients are derived as follows:
  (A23) $$c_{m}=\{\begin{array}{l} a_{m}+\sum_{k=1}^{m-1}\left\{\frac{k}{m}\right\}\cdot c_{k}\cdot a_{m-k}\left(1\leq m\leq Q\right)\\ \sum_{k=m-Q}^{m-1}\left\{\frac{k}{m}\right\}\cdot c_{k}\cdot a_{m-k}\left(m>Q\right) \end{array}$$

#### A.5. Long-Term Features

- Jitter is defined as:
  (A24) $$\text{Jitter}=\frac{\frac{1}{M-1}\sum_{i=1}^{M-1}\left|T_{i}-T_{i+1}\right|}{\frac{1}{M}\sum_{i=i}^{M}T_{i}}$$
  where *T_i_* is the estimated F0 value for frame *i* and *M* is the number of frames used for the calculation of Jitter.
- Shimmer is defined as:
  (A25) $$\text{Shimmer}=\frac{\frac{1}{M-1}\sum_{i=1}^{M-1}\left|A_{i}-A_{i+1}\right|}{\frac{1}{M}\sum_{i=i}^{M}A_{i}}$$
  where *A_i_* is the amplitude of frame *i* and *M* is the number of frames used for the calculation of shimmer.

### B. Surveyed Articles

In this overview, the following abbreviations are used for training methods:

- Gaussian mixture model (GMM)
- hidden Markov model (HMM)
- k-nearest neighbor (KNN)
- Linde-Buzo-Gray (LBG)
- Linde-Buzo-Gray-vector quantization (LBG-VQ)
- linear spectral pairs-vector quantization (LSP-VQ)
- neural networks (NN)
- support vector machine (SVM)
- vector quantization (VQ)
- weighted modified k-nearest neighbor (weighted D-KNN)

#### B.1. Global Sound Recognition

**Table:**

Article	Lu *et al.* (2002) [23]
Features	simple time, frequency, LPCC
Experiment, accuracy	speech: 97.00%, music: 93.00%, environment: 84.00%
Training method	KNN/LSP-VQ

Article	Nishimura and Kuroda (2008) [20]
Features	Haar-like
Experiment, accuracy	speech/non-speech: 96.93%
Training method	LBG-VQ

#### B.2. Age and Gender Recognition

**Table:**

Article	Nishimura (2012) [29]
Features	Haar-like
Experiment, accuracy	gender: 97.50%
Training method	LBG

Article	Ting *et al.* (2006) [44]
Features	frequency, MFCC
Experiment, accuracy	gender: 96.70%
Training method	GMM

Article	Zourmand *et al.* (2013) [45]
Features	frequency
Experiment, accuracy	gender: 97.50%
Training method	NN

Article	Pronobis and Magimai-Doss (2009) [24]
Features	frequency
Experiment, accuracy	gender: 100.00%
Training method	SVM

Article	Pronobis and Magimai-Doss (2009) [24]
Features	MFCC
Experiment, accuracy	gender: 97.90%
Training method	SVM

Article	Pronobis and Magimai-Doss (2009) [24]
Features	LPCC
Experiment, accuracy	gender: 97.20%
Training method	SVM

Article	Kim *et al.* (2007) [26]
Features	MFCC
Experiment, accuracy	age: 94.60%
Training method	GMM

Article	Kim *et al.* (2007) [26]
Features	MFCC
Experiment, Accuracy	gender: 94.90%
Training Method	GMM

Article	Kim *et al.* (2007) [26]
Features	MFCC
Experiment, accuracy	age + gender average: 88.90%
Training method	GMM

Article	Chen *et al.* (2011) [12]
Features	simple time, frequency, long-term
Experiment, accuracy	age + gender average: 51.40%
Training method	NN

Article	Chen *et al.* (2011) [12]
Features	simple time, frequency, long-term
Experiment, accuracy	male: 91.40%, female: 81.20%
Training method	NN

Article	van Heerden *et al.* (2010) [27]
Features	MFCC, long-term
Experiment, accuracy	age + gender average: 50.70%
Training Method	SVM

Article	Sadeghi Naini and Homayounpour (2006) [25]
Features	MFCC, long-term
Experiment, accuracy	gender: 86.50%
Training method	NN

Article	Sadeghi Naini and Homayounpour (2006) [25]
Features	MFCC, long-term
Experiment, accuracy	2 age classes: 72.00%, 3 age classes: 60.70%
Training method	NN

Article	Li *et al.* (2013) [46]
Features	simple time, frequency
Experiment, accuracy	4 age groups: 52.00%
Training method	GMM+SVM

Article	Li *et al.* (2013) [46]
Features	simple time, frequency
Experiment, accuracy	gender: 88.40%
Training method	GMM+SVM

#### B.3. Emotion Recognition

**Table:**

Article	Nogueiras *et al.* (2001) [39]
Features	simple time, frequency
Experiment, accuracy	7 emotions: 80.00%
Training method	HMM

Article	Nishimura (2012) [29]
Features	Haar-like
Experiment, accuracy	3 emotions: 84.60%
Training method	LBG

Article	Nwe *et al.* (2003) [36]
Features	Frequency
Experiment, accuracy	6 emotions: 78.10%
Training method	HMM

Article	Busso *et al.* (2009) [38]
Features	frequency
Experiment, accuracy	15 emotions: 77.00%
Training method	GMM

Article	He *et al.* (2009) [35]
Features	frequency
Experiment, accuracy	stress detection: 81.00%
Training method	GMM

Article	Bou-Ghazale and Hansen (2000) [47]
Features	MFCC
Experiment, accuracy	stress detection, 4 levels: 83.66%
Training method

Article	Neiberg *et al.* (2006) [34]
Features	frequency, MFCC
Experiment, accuracy	3 emotions: 90.00%
Training method	GMM

Article	Pao *et al.* (2006) [37]
Features	MFCC, LPCC
Experiment, accuracy	6 emotions: 79.55%
Training method	weighted D-KNN

Article	Tosa and Nakatsu (1996) [48]
Features	LPCC
Experiment, accuracy	7 emotions: 60.00%
Training method	ANN

Article	Ooi *et al.* (2014) [49]
Features	simple time, MFCC
Experiment, accuracy	6 emotions: 75.90%
Training method	NN

Article	Giannoulis and Potamianos (2012) [50]
Features	simple time, frequency, MFCC
Experiment, accuracy	6 emotions: 85.18%
Training method	SVM based

#### B.4. Person Recognition

**Table:**

Article	Alpert and Allen (2010) [13]
Features	simple time
Experiment, accuracy	upstairs: 82.87%, downstairs: 87.59%
Training method	NN

Article	Nishimura (2012) [29]
Features	Haar-like
Experiment, accuracy	identification, 12 speakers: 93.00%
Training method	LBG

Article	Kinnunen *et al.* (2008) [28]
Features	Frequency
Experiment, accuracy	verification, 170 target speakers: 82.60%
Training method

Article	Kinnunen *et al.* (2008) [28]
Features	MFCC
Experiment, accuracy	verification, 170 target speakers: 92.70%
Training method

Article	Hasan *et al.* (2004) [30]
Features	MFCC
Experiment, accuracy	identification, 24 speakers: 100.00%
Training method	VQ

Article	Tiwari (2010) [32]
Features	MFCC
Experiment, accuracy	verification, 5 speakers: 85.00%
Training method	VQ

Article	Reynolds *et al.* (2000) [33]
Features	MFCC
Experiment, accuracy	verification, 11 speakers: 90.00%
Training method	GMM

Article	Murty and Yegnanarayana (2006) [51]	
Features	MFCC	
Experiment, accuracy	verification, 149 male speakers: 86.00%	
Training method	NN	

Article	Murty & Yegnanarayana (2006) [51]	
Features	LPCC	
Experiment, accuracy	verification, 149 male speakers: 78.00%	
Training method	NN	

Article	Murty & Yegnanarayana (2006) [51]	
Features	MFCC, LPCC	
Experiment, accuracy	verification, 149 male speakers: 89.50%	
Training method	NN	

Article	Kim *et al.* (2004) [31]	
Features	MFCC	
Experiment, accuracy	identification, 195 speakers: 95.45%	
Training method	GMM	

#### B.5. Indoor and Outdoor Environment Recognition

**Table:**

Article	Stäger *et al.* (2004) [17]
Features	simple time, frequency
Experiment, accuracy	5 kitchen sounds: 85.00%, 5 workshop sounds: 67.00%
Training method	C4.5 decision tree/3NN

Article	Nishimura (2012) [29]
Features	Haar-like
Experiment, accuracy	21 sounds: 97.30%
Training method	LBG

Article	Zhan (2012) [40]
Features	Haar-like
Experiment, accuracy	20 sounds: 96.00%
Training method	HMM

Article	Chen *et al.* (2005) [16]
Features	MFCC
Experiment, accuracy	6 bathroom sounds: 83.50%
Training method	HMM

Article	Sehili *et al.* (2012) [52]
Features	MFCC
Experiment, accuracy	18 indoor sounds: 75.00%
Training method	SVM

Article	Guo *et al.* (2012) [53]
Features	long-term
Experiment, accuracy	10 indoor sounds: 92.00%
Training method	NN

Article	Park *et al.* (2014) [54]
Features	MFCC
Experiment, accuracy	9 events: 91.00%
Training method	GMM

Article	Rabaoui *et al.* (2009) [55]
Features	simple time, frequency, MFCC
Experiment, accuracy	9 surveillance sounds: 93.00%
Training method	HMM

Article	Łopatka *et al.* (2010) [18]
Features	simple time, frequency
Experiment, accuracy	5 danger sounds: 97.07%
Training method	SVM

Article	Peltonen *et al.* (2002) [19]
Features	frequency
Experiment, accuracy	17 environment sounds: 63.40%
Training method	1NN

Article	Peltonen *et al.* (2002) [19]
Features	MFCC
Experiment, accuracy	17 environment sounds: 63.40%
Training method	GMM

Article	Krijnders *et al.* (2010) [56]
Features	frequency
Experiment, accuracy	21 sounds: 42.00%
Training method	knowledge network

Article	Couvreur *et al.* (1998) [57]
Features	LPCC
Experiment, accuracy	5 noise events: 90.00%
Training method	HMM

Article	Heittola *et al.* (2013) [58]
Features	MFCC
Experiment, accuracy	10 outdoor contexts: 91.00%
Training method	GMM
